# Headwater gas exchange quantified from O_2_ mass balances at the reach scale

**DOI:** 10.1002/lom3.10281

**Published:** 2018-09-28

**Authors:** L. Rovelli, K. M. Attard, C. M. Heppell, A. Binley, M. Trimmer, R. N. Glud

**Affiliations:** ^1^ Scottish Marine Institute Scottish Association for Marine Sciences Oban United Kingdom; ^2^ Nordcee, Department of Biology University of Southern Denmark Odense M Denmark; ^3^ Tvärminne Zoological Station University of Helsinki Hanko Finland; ^4^ School of Geography Queen Mary University of London London United Kingdom; ^5^ Lancaster Environment Centre Lancaster University Lancaster United Kingdom; ^6^ The School of Biological and Chemical Sciences Queen Mary University of London London United Kingdom; ^7^ Department of Ocean and Environmental Sciences Tokyo University of Marine Science and Technology Tokyo Japan

## Abstract

Headwater streams are important in the carbon cycle and there is a need to better parametrize and quantify exchange of carbon‐relevant gases. Thus, we characterized variability in the gas exchange coefficient (*k*_2_) and dissolved oxygen (O_2_) gas transfer velocity (*k*) in two lowland headwaters of the River Avon (UK). The traditional one‐station open‐water method was complemented by in situ quantification of riverine sources and sinks of O_2_ (i.e., groundwater inflow, photosynthesis, and respiration in both the water column and benthic compartment) enabling direct hourly estimates of *k*_2_ at the reach–scale (~ 150 m) without relying on the nighttime regression method. Obtained *k*_2_ values ranged from 0.001 h^−1^ to 0.600 h^−1^. Average daytime *k*_2_ were a factor two higher than values at night, likely due to diel changes in water temperature and wind. Temperature contributed up to 46% of the variability in *k* on an hourly scale, but clustering temperature incrementally strengthened the statistical relationship. Our analysis suggested that *k* variability is aligned with dominant temperature trends rather than with short‐term changes. Similarly, wind correlation with *k* increased when clustering wind speeds in increments correspondent with dominant variations (1 m s^−1^). Time scale is thus an important consideration when resolving physical drivers of gas exchange. Mean estimates of *k*_600_ from recent parametrizations proposed for upscaling, when applied to the settings of this study, were found to be in agreement with our independent O_2_ budget assessment (within < 10%), adding further support to the validity of upscaling efforts aiming at quantifying large‐scale riverine gas emissions.

Revisions of the global carbon cycle have indicated that rivers and other inland waters contribute substantially to the global cycling of organic carbon and emission of carbon dioxide (CO_2_) and methane (CH_4_) to the atmosphere (e.g., Cole et al. 2007; Battin et al. 2008; Tranvik et al. 2009; Aufdenkampe et al. 2011; Raymond et al. 2013; Hotchkiss et al. 2015; Stanley et al. 2015; Marx et al. 2017). However, due to a lack of an appropriate universal scaling for quantification of emissions, headwaters (stream order < 4) were initially not included in these assessments (*see* Cole et al. 2007), despite the fact they represent 17% of global riverine area (in perennial riverine systems e.g., Downing et al. 2012). Later inclusions of headwaters have proposed that small streams (order 1) might represent more than a third of the total regional emissions of CO_2_ from riverine systems (Butman and Raymond 2011) and thus could be more prominent at the global scale (Raymond et al. 2013). This has highlighted the need for better constraining gas exchange at the reach scale (Trimmer et al. 2012), with the overarching goal of fine‐tuning parametrizations used for large scale and global upscaling of metabolism and gas emissions.

Accurate assessments of gas exchange require quantification of the gas transfer velocity, or piston velocity (*k;* unit of length per unit of time^−1^, e.g., m h^−1^), which physically controls the exchange of gases at the stream–air interface (*see* Hall et al. 2012). Thus, estimates of *k* are critical to the assessment of emission of greenhouse gases such as CO_2_ (e.g., Hotchkiss et al. 2015; Marx et al. 2017) and CH_4_ (e.g., Stanley et al. 2015; Crawford and Stanley 2016) and for the quantification of the gas exchange rate of dissolved oxygen (O_2_) and estimates of whole‐reach metabolism (e.g., Odum 1956). Stream gas exchange is most commonly assessed from total, i.e., whole‐stream, metabolism estimates using open water (OW) methods based on local dissolved oxygen (O_2_) mass balances (e.g., Odum 1956; Marzolf et al. 1994; Mulholland et al. 2001; Riley and Dodds 2013; Siders et al. 2017). The OW approach provides an integrative quantification of gas exchange *R*_ex_ (in *μ*mol L^−1^ unit of time^−1^) as

(1) $$R_{\mathrm{ex}}=k_{2}\left(O_{2\left(\mathrm{sat}\right)}-O_{2}\right)$$

with (O_2(sat)_ ‐ O_2_) being the O_2_ saturation deficit, i.e., the difference between the O_2_ concentration at saturation for the local physical conditions (O_2(sat)_) and the actual O_2_ concentration in the stream water, and *k*_2_ the O_2_ gas exchange coefficient, often termed the re‐aeration coefficient (*k*_2_ or *K*, unit of time^−1^, e.g., h^−1^). The coefficient *k*_2_ represents the product of the depth‐corrected O_2_ gas transfer velocity, or piston velocity (*k;* unit of length per unit of time^−1^, e.g., m h^−1^) and local stream area‐to‐volume ratio, which is often approximated as the inverse of the mean water depth (in m^−1^) (*see* Raymond et al. 2012; Demars et al. 2015). As assessments of gas exchange critically rely on the quantification of *k*_2_ or *k*, several direct and indirect methods have been developed for deriving these values.

Direct measurements of these parameters are characterized by the de‐gassing of a conservative tracer gas that is subsequently scaled to O_2_ (e.g., Wanninkhof et al. 1990; Genereux and Hemond 1992; Reid et al. 2007; Benson et al. 2014). This approach is logistically demanding and temporal upscaling is difficult (Demars et al. 2015). Indirect measurements depend on parameterizations of the local physical characteristics of the stream reach such as depth, flow and slope (e.g., Parker and Gay 1987; Parker and DeSimone 1992 and references therein), as well as fitting of gas transfer parameters to O_2_ time series based on simplified relationships (Hornberger and Kelly 1975; Chapra and DiToro 1991; McBride and Chapra 2005) or more complex modeling efforts (Holtgrieve et al. 2010; Grace et al. 2015; Appling et al. 2018). Empirical equations and parameterizations have proven to be useful as predictors of gas‐exchange dynamics for specific reaches (*see* Genereux and Hemond 1992), but usually show large discrepancies in *k*_2_ estimates when applied to streams with comparable hydrological characteristics (Moog and Jirka 1998; Aristegi et al. 2009; Palumbo and Brown 2014). To further constrain these parametrizations for spatial upscaling, recent studies have focused on a better characterization of the stream morphology. For example, Raymond et al. (2012) used a large collection of stream metadata to more rigorously scale *k* parametrizations to stream physical characteristics (i.e., flow, depth, slope, discharge).

Although *k* variability on short timescales, i.e., hours, has been reported (e.g., Tobias et al. 2009; Berg and Pace 2017), direct and indirect methods mostly focus on determining a mean value for *k*_2_ for either the day or night, or an average for the whole day‐night period, with little consideration being given to its short‐term dynamics that are characteristic of most rivers. Recent application of the aquatic eddy co‐variance technique (AEC) in rivers has provided robust assessment of reach‐scale (~ 150 m) benthic metabolism (Koopmans and Berg 2015; Rovelli et al. 2017) and, most recently, an “inverted” AEC approach has been used to directly quantify O_2_ gas exchange in large (order 3) streams (Berg and Pace 2017) and in an estuarine embayment (Long and Nicholson 2018). Yet, irrespective of the approach being applied to assess *k*, little emphasis has been given to small headwaters streams (order 1–2), despite their potential implications for the regional and global carbon cycling (e.g., Butman and Raymond 2011; Raymond et al. 2013).

The goal of our study was to provide a proof‐of‐concept for the derivation (values and dynamics) of *k*_2_ and *k* at the reach scale in small, headwater streams (stream order 1). This was achieved by combining in situ O_2_ measurements from a single station OW approach together with direct assessments of riverine metabolism using AEC, sample incubations, and groundwater inflow measurements. The proposed method was compared to the traditional nighttime regression (NR) method, and was used to characterize temporal variability in *k* on hourly to daily time scales. Furthermore, we evaluate *k* dynamics and its relation to the local physical drivers such as mean stream flow, temperature and wind patterns. The results of this study are discussed in light of parametrizations of *k* for regional to large‐scale upscaling.

## *Materials and procedures*

### Study site

This study focused on two headwaters of the Hampshire River Avon, southern England: the Chalk River Ebble (CE) and the Greensand West Avon (GA) (*see* Heppell et al. 2017; Supporting Information Fig. S1). For each headwater, a representative ~ 150 m reach was selected based on previous surveys of the sub‐catchment morphology and geology (*see* “Field measurements” section). The selected CE reach (CE1^1^; 51° 1′ 41.171″ N/1° 54′ 56.309″ W) was investigated over 3 d (25–27 April 2013) in spring, when the reach was characterized by a net outflow of stream water to the aquifer, i.e., a losing reach (*see* Supporting Information Fig. S2). The reach was also characterized by profuse growth (36% cover by area) of *Ranunculus* spp., which is widespread throughout the sub‐catchment (Watson 1987). The water column of the River Ebble was characterized by low turbidity, with suspended sediment concentration limited to, on average, 37 mg L^−1^ (Heppell and Binley 2016a). The selected GA reach (GA2; 51° 19′ 10.173″ N/1° 51′ 33.135″ W) was investigated over 3 d from 28–30 April 2013. In contrast to the CE reach, the GA reach was characterized by a constant inflow of low‐oxygenated groundwater; i.e., a gaining reach (Supporting Information Fig. S2) and only patchy macrophyte coverage (4% by area). Turbidity in the water column was 40% higher on average than at the CE reach (Heppell and Binley 2016a). The reaches are referred to as CE and GA throughout the text.

### Reach scale oxygen budget

The assessment of O_2_ gas exchange and stream metabolism based on a single station open‐water approach (e.g., Odum 1956) relies on O_2_ time series. Temporal changes in O_2_ concentration, *d*O_2_/*dt*, are attributed to local whole‐stream primary production (*P*), respiration (*R*) and atmospheric exchange (*R*_ex_) as

(2) $$\frac{{dO}_{2}}{\mathrm{dt}}=P+R+R_{\mathrm{ex}}=P+R+k_{2}\left(O_{2\left(\mathrm{sat}\right)}-O_{2}\right)$$

with *k*_2_ being the gas exchange coefficient (in h^−1^) and (O_2(sat)_ − O_2_) the O_2_ saturation deficit. The spatial integration of a one‐station approach, i.e., the integrated stretch length along the stream, is typically in the order of 1000s of m and can be quantify as 3*v*/*k*_2_, with *v* being the mean stream flow velocity (in m d^−1^) and *k*_2_ (in d^−1^) (*see* Grace and Imberger 2006).

Rates for *R* and *R*_ex_ are typically estimated by applying the established nighttime regression (NR) method (Hornberger and Kelly 1975). The method plots the nighttime *d*O_2_/*dt* at each time step against the O_2_ saturation deficit during the night and assumes *P* = 0 and that respiration is light‐independent. The resulting linear relationship, obtained via least‐squares regression, provides both *k*_2_ (slope) and *R* (intercept). As NR‐based *k*_2_, *k*_NR_, is only obtained at night, many studies have adopted a temperature correction term to account for temperature changes between night and day following the parameterization of Elmore and West (1961):

(3) $$k_{2\left(T\right)}=k_{2\left(20{}^{\circ}C\right)}{\left(1.0241\right)}^{\left(T-20\right)}$$

where *k*_2(20°C)_ is the gas exchange coefficient at 20°C and *k*_2(*T*)_ the gas exchange coefficient at the given temperature *T*.

**Figure 1:**
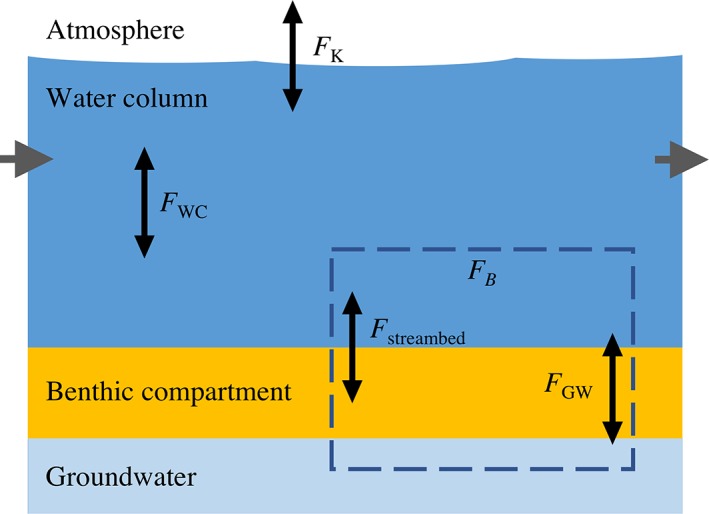
Dissolved oxygen mass balance approach. Changes in stream O_2_ concentration are expressed as a function of contributions from the water column (*F*_WC_), benthic compartment (*F*_B_), and atmospheric exchange (*F*_K_) following the parameterization of Eq. (4). Benthic *F*_B_ includes contributions from the streambed (*F*_streambed_) and from groundwater inflow/outflow (*F*_GW_). Note that lateral exchange and advection processes were not considered.

The O_2_ budget (OB) approach used in the current study expands Eq. (2) by accounting for all relevant processes including (1) metabolic activity in both sediment and water, and (2) exchange with both the atmosphere and groundwater (Fig. 1). The temporal O_2_ concentration was thus defined as:

(4) $$\frac{{dO}_{2}}{\mathrm{dt}}=\frac{A}{V}\left[F_{B}+F_{\mathrm{WC}}+F_{K}\right]$$

where *F*_B_, *F*_WC_, *F*_K_ are the O_2_ flux (in mmol m^−2^ h^−1^) associated with the benthic compartment, the water column, and atmospheric exchange, respectively. The O_2_ fluxes were expressed as volumetric rates (in *μ*mol L^−1^ h^−1^) by multiplying the values by the average ratio between stream area (A) and stream volume (V), which, under smooth stream water surface conditions, is approximated as the mean stream depth (*d*) (Demars et al. 2015).

The main strength of the proposed approach is that each flux component within the OB (Eq. (4)) can be directly quantified in the field. Estimates of *F*_B_ were obtained from aquatic eddy co‐variance (AEC) measurements, while *F*_WC_ was quantified by incubating discrete water samples in situ (*see* “Field measurements” section). While *F*_B_ does include both streambed (*F*_streambed_) and groundwater contributions to the O_2_ budget, fluxes associated with the inflow of O_2_ depleted groundwater were independently quantified as:

(5) $$F_{\mathrm{GW}}=v_{\mathrm{gw}}\left(O_{2\left(\mathrm{gw}\right)}-O_{2}\right)$$

with O_2(gw)_ being the groundwater O_2_ concentration (in *μ*mol L^−1^), O_2_ the in‐stream concentration and *v*_gw_ the local groundwater inflow (in m h^−1^).

The atmospheric exchange rate was quantified as:

(6) $$F_{K}=\frac{V}{A}k_{2}\left(O_{2\left(\mathrm{sat}\right)}-O_{2}\right)$$

with *k* = *k*_2_(*V*/*A*) ≈ *k*_2_ *d* being the O_2_ gas transfer velocity, or O_2_ piston velocity (in m h^−1^) and O_2(sat)_ the local O_2_ concentration at saturation. As O_2(sat)_ can be quantified as a function of temperature (*see* Garcia and Gordon 1992), the OB approach effectively enabled a direct estimate of *k*_2_.

To facilitate comparisons with published reference values and parameterizations from previous studies, *k*_2_ values were also expressed as *k*_2(20°C)_ (*see* Eq. (3)). Similarly, estimates of O_2_ gas transfer velocity were also standardized to a Schmidt number of 600^2^ (*k*_600_) based on Jahne et al. (1987) as

(7) $$\frac{k_{600}}{k}={\left(\frac{600}{{\mathrm{Sc}}_{T}}\right)}^{-1/2}$$

where *Sc*_*T*_ ≈ 0.00086842*T*^4^ − 0.10115*T*^3^ + 4.8055*T*^2^ − 124.34*T* + 1745.1 is the Schmidt number for O_2_ in freshwater at the local water temperature *T* (*see* Wanninkhof 2014). Stream temperature time series were obtained from background measurements of physical parameters during the observational period at each site (Rovelli et al. 2016b).

### Field measurements

#### *Site selection*

The investigated reaches (CE and GA) were carefully selected to represent the sub‐catchment dominant stream morphology features (e.g., stream course shape, depth, bends, riparian zone) and in‐stream habitat patchiness (macrophytes, sediment type). Particular emphasis was given to ensure that each reach also fulfilled the theoretical requirements of the NR method, i.e., streams with relatively high metabolic activity and low gas exchange (*see* Hornberger and Kelly 1975). Field measurements were also executed under stable hydrological condition, i.e., constant flow and mean water depth over ≥ 3 d. This enabled (1) the evaluation of our local O_2_ budget (OB) method and (2) comparisons with the nighttime regression (NR) method and standardized functional equations for *k*_2_ quantification from mean hydrological parameters.

#### *Background physicochemical measurements*

Stream water column O_2_ concentration, temperature, and photosynthetically active radiation (PAR) at the streambed (~ 15 cm above the bottom) were monitored within each reach at 1 min intervals with a conductivity–temperature–depth logger (CTD; XR‐420, RBR, Kanata, Canada), equipped with an Aanderraa O_2_ optode sensor (Aanderaa, Bergen, Norway) and a 4π PAR sensor (QCP‐2000; Biospherical Instruments, San Diego, U.S.). The data were also used to calibrate the O_2_ sensors used for flux estimates and to define nighttime periods (PAR < 2 *μ*mol quanta m^−2^ s^−1^). Wind speed and direction near the stream surface (1.5 m elevation) were recorded at 15 s intervals with a SkyWatch GEOS 11 portable weather station (JDC Electronic SA, Yverdon‐les‐Bains, Switzerland).

#### *Benthic oxygen flux*

The benthic oxygen flux was quantified using the AEC technique (Berg et al. 2003). Our AEC module consisted of small, lightweight, stainless steel tripod frame equipped with an acoustic Doppler velocimeter (ADV; Vector, Nortek A/S, Rud, Norway), Clark‐type O_2_ microelectrodes (Revsbech 1989), and submersible O_2_ amperometric amplifiers (McGinnis et al. 2011). Up to two O_2_ microelectrodes were positioned ~ 0.5 cm outside the ADV sampling volume, at an inclination of ~ 60°. Each microelectrode had a 90% response times < 0.5 s and stirring sensitivity < 0.5% (Gundersen et al. 1998). The ADV's flow measurements were obtained at 8–15 cm from the streambed with a frequency of 64 Hz and with the ADV *x*‐axis aligned to the main flow direction. The acquired datasets were processed following the same procedure described in Rovelli et al. (2017). In short, the dataset was averaged to 8 Hz while applying data quality controls and despiking routines (e.g., Mori et al. 2007) to the dataset to remove dataset artifacts and reduce signal noise. A double coordinate rotation was applied to the flow time series to minimize the influence of horizontal motions on the vertical velocity component. The datasets were then aligned, i.e., time‐shifted, to account for the relative distance between the ADV sampling volume and the microelectrode tip and for the microelectrode time constant (*see* Donis et al. 2015). The AEC‐based turbulent oxygen fluxes (in mmol m^−2^ h^−1^) were estimated from time‐averaged vertical velocity fluctuations (*w*′) and O_2_ concentration fluctuations (*C*′), as

(8) $$F_{\mathrm{AEC}}=\bar{w\prime C\prime }$$

(*see* Berg et al. 2003) via a Reynolds’ decomposition, specifically linear detrending, using the program suite Sulfide‐Oxygen‐Heat Flux Eddy Analysis (SOHFEA version 2.0; *see* McGinnis et al. 2014). The optimal detrending interval represents a trade‐off between including low‐frequency turbulent contributions and excluding non‐turbulent contributions (McGinnis et al. 2008), and was inferred to be 5 min from cumulative averages of oxygen fluxes and friction velocity (u_*_) (*see* Attard et al. 2014). To account for the effect of transient O_2_ concentration changes in the water column between the sediment–water interface and the AEC measurement height (*h*), which can potentially bias *F*_AEC_ (*see* Holtappels et al. 2013; Rheuban et al. 2014), an O_2_ storage term was estimated after Rheuban et al. (2014). The benthic oxygen flux corrected for O_2_ storage, *F*_B_, was defined as

(9) $$F_{B}=F_{\mathrm{AEC}}+F_{\text{storage}}=F_{\mathrm{AEC}}+\int_{0}^{h}\frac{\mathrm{dC}}{\mathrm{dt}}\mathrm{dz}$$

with *dC*/*dt* being the measured O_2_ concentration gradient.

The smallest area of the streambed that contributes to 90% of AEC flux, termed the “footprint area”, was estimated from the sediment surface roughness parameter (*z*_0_) and *h* following the parameterization by Berg et al. (2007). Values for *z*_0_ were approximated as $z_{0}=h\cdot \exp\left(-\kappa \cdot \frac{u}{u_{*}}\right)$ with *κ* being the von Karman constant (0.41), and *u* the average flow velocity (Wüest and Lorke 2003).

#### *Water column activity*

Water column oxygen production and consumption rates were estimated by incubating 100 mL rack‐mounted glass bottles in situ over 24 h in the light and in the dark, with continuous measurements of O_2_ concentration via O_2_ optical fibers (4 channel FirestingO2; Pyro Science GmbH, Aachen, Germany). A set of one dark bottle, and one clear bottle were incubated at the streambed, while one bottle was incubated near the stream surface. The fourth bottle was either incubated at mid‐depth at GA (depth > 0.4 m) or near the surface for the shallower CE. Three additional replicate sets were also mounted on the rack but O_2_ measurements were only performed at the start and end of each incubation via Winkler titrations (Winkler 1888). Volumetric oxygen fluxes (in *μ*mol L^−1^ h^−1^) were estimated from temporal O_2_ concentration gradients via linear regressions for daytime and nighttime periods, respectively. No difference in fluxes were observed at the different depths, indicating a homogenously, well‐irradiated water column and thus depth‐independent rates. The areal oxygen fluxes *F*_WC_ (in mmol m^−2^ h^−1^) were obtained by multiplying the values with the average stream depth. Contributions from macrophytes were deemed to be well‐integrated within benthic measurements and thus were not further addressed within the water column oxygen budget (Rovelli et al. 2017).

#### *Groundwater influx*

Dissolved oxygen flux resulting from local inflow of O_2_ depleted groundwater was estimated based on measurements of the local hydraulic gradients from in‐stream piezometers and porewater O_2_ concentrations. Two clusters of in‐stream piezometers were installed at each site, ~ 10 m apart in the river thalweg. Each cluster comprised three piezometers screened at 20 cm, 50 cm, and 100 cm depth; the 100 cm piezometer was also fitted with narrow polytetrafluoroethylene tubing to depths of 10 cm, 20 cm, 30 cm, 50 cm, 70 cm, and 100 cm for the purposes of pore water sampling. Installation and design of the piezometers followed the description in Binley et al. (2013).

Hydraulic head was measured in all piezometers using HOBO pressure transducers (Onset Computer Corporation, Bourne, U.S.) at GA and Levelogger Edge pressure transducers (Solinst, Georgetown, Canada) at CE. Measurements were validated with manual dips on a fortnightly basis and, if needed, corrected assuming a linear drift. River stage was measured using a pressure transducer suspended within a stilling well. Falling and rising head slug tests (measurements taken by pressure transducers installed inside the piezometers) were used for computation of saturated hydraulic conductivity using the Hvorslev method (e.g., Binley et al. 2013). Vertical groundwater flux was computed using Darcy's Law, from measurements of the hydraulic gradient between the 50 cm deep piezometric head and local stream stage, combined with a weighted harmonic mean of hydraulic conductivity from slug tests carried out in the 20 cm and 50 cm deep piezometers (*see* Binley et al. 2013).

Pore water samples were collected from sampling tubes located on the piezometers every 2 months, from February 2014 to June 2016, using a syringe and tygon tubing. The O_2_ concentration of pore water and river water was measured immediately following sample collection using a calibrated Clark‐type O_2_ microelectrode (50 *μ*m tip) connected to an in‐line amplifier and data‐logging meter (Unisense, Aarhus, Denmark) (*see* Heppell and Binley 2016b).

#### *Data handling*

Collected hydrological and physicochemical data were used to (1) characterize average conditions at each reach and (2) assess short term, i.e., hourly, to diel dynamics. O_2_ fluxes obtained for each stream compartment were considered separately, to assess their magnitude and variability within and across the reaches, as well as within the O_2_ budget context, to quantify and characterize *k* variability.

## *Assessment*

### Reach characterization

**Figure 2:**
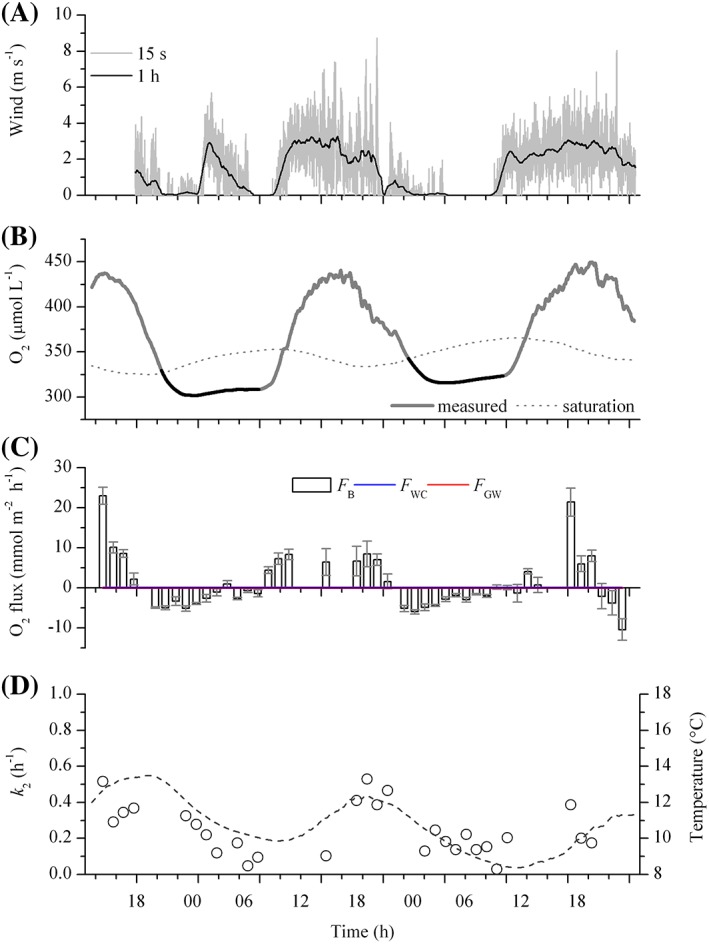
Estimates of *k*_2_ for the River Ebble (CE) during the spring. (**A**) Wind speed recorded 1.5 m above the stream surface. (**B**) Local dissolved oxygen (O_2_) concentration (gray line) and associated O_2_ concentration at atmospheric saturation (dotted line). Black lines indicate nighttime (PAR < 2 *μ*mol quanta m^−2^ s^−1^). (**C**) Time series of oxygen fluxes encompassing benthic (*F*_B_), water column (*F*_WC_), and groundwater (*F*_GW_) contributions to the local O_2_ budget. (**D**) Hourly averaged estimates of *k*_2_ based on the O_2_ budget (OB) method in spring (circles), with temperature overlain (dashed line).

**Figure 3:**
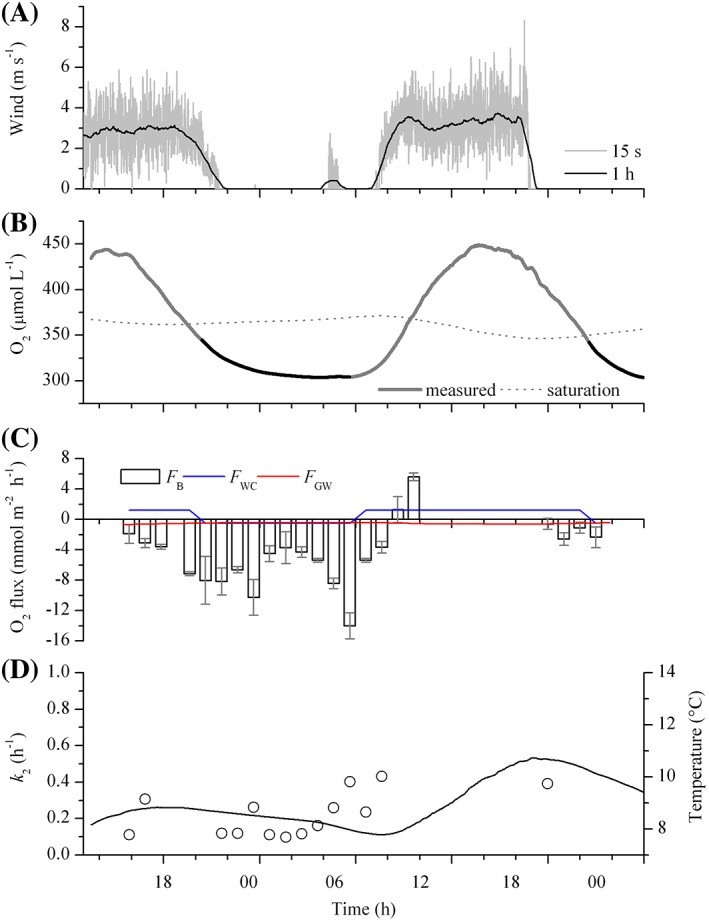
Estimates of *k*_2_ for the river West Avon (GA) during the spring. (**A**) Wind speed recorded 1.5 m above the stream surface. (**B**) Local dissolved oxygen (O_2_) concentration (gray line) and associated O_2_ concentration at atmospheric saturation (dotted line). Black lines indicate nighttime (PAR < 2 *μ*mol quanta m^−2^ s^−1^). (**C**) Time series of oxygen fluxes encompassing benthic (*F*_B_), water column (*F*_WC_), and groundwater (*F*_GW_) contributions to the local O_2_ budget. (**D**) Hourly averaged estimates of *k*_2_ based on the O_2_ budget (OB) method in spring (circles), with temperature overlain (solid line). Note that the wind data were shifted by 24 h to fill the measurement gap on day 1.

Hydrological data and background information for each reach are summarized in Table 1. Temperature ranged from 7.8°C to 13.5°C overall, reflecting a moderate variation (< 5%) between day and night at the two sites. Daily integrated PAR (PAR_24_) ranged between 11.9 mol quanta m^−2^ d^−1^ and 17.4 mol quanta m^−2^ d^−1^, with peaks of up to 1630 *μ*mol quanta m^−2^ s^−1^. Overall, the average stream flow, based on ADV measurements (12 cm above the streambed), was in the order of 0.18–0.33 m s^−1^ with an associated discharge of 0.209–0.640 m^3^ s^−1^ (Table 1) and showed no significant change between day and night, or within the observational period, indicating a stable flow. Wind speeds at CE showed irregular patterns but an overall higher wind speed during the day than at night (Fig. 2). Wind dynamics at GA, in contrast, were markedly diel, with an average wind speed of 2.4–3 m s^−1^ during the day and 0–0.2 m s^−1^ at night (Fig. 3). At both sites the water column showed well‐defined diel fluctuations in O_2_ concentration, with nighttime under‐saturation (down to 82%) and daytime oversaturation (up to 133%), with diel O_2_ concentration changes reaching ~ 147 *μ*mol L^−1^ (Figs. 2, 3). The O_2_ saturation deficit ranged from −108 to −96 *μ*mol L^−1^ during the day, to 43–65 *μ*mol L^−1^ at night. The average deficit over 24 h ranged from 2 *μ*mol L^−1^ to an O_2_ saturation surplus of 15 *μ*mol L^−1^.

**Table 1:** Summary of hydrological and physicochemical data for river Ebble (CE) and West Avon (GA). Average values are presented as mean ± standard error. Values between square brackets indicate the range of the measurements.

Site	Period	Daylight [h]	Daily PAR [mol m^‐2^ d^−1^]	O_2_ [% sat]	O_2_ [*μ*mol L^−1^]	T [°C]	Depth^a^ [m]	Width^a^ [m]	Flow [m s^−1^]	Discharge^a^ [m^3^ s^−1^]
CE	25–27 Apr 2013	15.3	11.9	106.6±0.3 [87.7–132.7]	367.7±0.9 [301.5–449.5]	10.8±0.03 [8.4–13.5]	0.41	5.25	0.18	0.640
GA	28–30 Apr 2013	15.3	17.4	101.5±0.3 [82.3–127.2]	365.3±1.2 [303.0–449.3]	9.0±0.02 [7.8–10.7]	0.57	2.75	0.33	0.385

a Obtained from depth and flow velocity transects performed during the observational period under stable hydrograph (*see* Rovelli et al. 2016a).

### Dissolved oxygen budget

#### *Benthic compartment*

Hourly oxygen fluxes for the benthic compartment ranged between −14.0 mmol m^−2^ h^−1^ and 23.0 mmol m^−2^ h^−1^ (Figs. 2, 3). At CE, oxygen fluxes followed a clear diel pattern (Fig. 2), with average daytime rates of 4.2 mmol m^−2^ h^−1^ and nighttime rates of −2.8 mmol m^−2^ h^−1^. AEC‐based benthic fluxes were estimated to cover a footprint area 26 m long and 1 m wide (~ 20 m^2^), with most of the flux contribution, i.e., the region of maximum flux, located 0.5 m upstream of the AEC sampling point. At GA, a diel pattern in the benthic oxygen fluxes was also observed (Fig. 3). However, both mean fluxes at night and during the day were negative, amounting to −6.2 mmol m^−2^ h^−1^ and −3.0 mmol m^−2^ h^−1^, respectively. The theoretical footprint area was 80 m long and 1 m wide (~ 70 m^2^) with the region of maximum flux located 2 m upstream of the of the AEC sampling point.

#### *Water column*

At CE, metabolic activity in the water column was below detection (< 0.1 *μ*mol L^−1^ h^−1^) and negligible for the local O_2_ budget (Fig. 2). In contrast, in the more turbid, sandy GA, water column activity ranged from net oxygen production rates of 2.8 *μ*mol L^−1^ h^−1^ during the day, to oxygen consumption of −0.9 *μ*mol L^−1^ h^−1^ at night. The average daytime rate of oxygen production was ~ 1.2 mmol m^−2^ h^−1^, with an average nighttime consumption rate of −0.5 mmol m^−2^ h^−1^ (Fig. 3).

#### *Groundwater*

During our study, the CE reach was losing water to the aquifer. This net local outflow of stream water had no measurable effect on in‐stream O_2_ concentrations (Fig. 2). At gaining reach GA, the inflow of groundwater amounted to 0.043 m d^−1^ on average, with a mean groundwater O_2_ concentration of 63.5 *μ*mol L^−1^. Groundwater contributed to an areal O_2_ concentration decrease of −0.4 mmol m^−2^ h^−1^ to −0.7 mmol m^−2^ h^−1^ (average −0.5 mmol m^−2^ h^−1^), representing 6–7% of the combined nighttime flux caused by metabolic activity in the benthic compartment and the water column (Fig. 3).

#### *Atmospheric exchange*

The derived rate of O_2_ exchange between the stream and the atmosphere ranged from −22.3 mmol m^−2^ h^−1^ to 14.5 mmol m^−2^ h^−1^. Estimates of *k*_2_ based on the O_2_ budget (OB) method ranged from 0.001 to 0.600 h^−1,^ with mean values of 0.252 h^−1^ and 0.259 h^−1^ for CE and GA, respectively (Table 2). Within such range of *k*_2_ and with mean flow velocities of 0.18–0.33 m s^−1^, one‐station based assessment are expected to integrate a stream length of > 7 km).

**Table 2:** Estimates of *k*_2_ (in h^−1^) using the O_2_ budget (OB) method. Values for daytime, nighttime, and average over the observational period are reported as mean ± standard error, with *n* indicating the number of averaged data points. Standardized *k*_2(20°C)_ (in h^−1^) and *k*_600_ (in m h^−1^) are also reported to enable better comparisons with literature studies.

Site	Day	Night	Mean
	*k*_*2*_ [h^−1^] (*n*)		
CE	0.331±0.024 (63)	0.170±0.012 (62)	0.252±0.015 (125)
GA	0.367±0.034 (26)	0.188±0.020 (37)	0.261±0.021 (63)
	*k*_2(20°C)_ [h^−1^] (*n*)		
CE	0.404±0.030 (63)	0.214±0.014 (62)	0.310±0.019 (125)
GA	0.478±0.045 (26)	0.248±0.027 (37)	0.343±0.028 (63)
	*k*_600_ [m h^−1^] (*n*)		
CE	0.157±0.012 (63)	0.084±0.006 (62)	0.121±0.007 (125)
GA	0.265±0.025 (26)	0.137±0.015 (37)	0.190±0.016 (63)

Estimates of the gas exchange coefficient *k*_2_ using the NR method (*k*_NR_) were 0.555 h^−1^ at CE and 0.434 h^−1^ at GA (Table 3; Supporting Information Fig. S3), and thus around a factor 2 higher than those obtained with the OB method. (Tables 2, 3). At CE, we observed a significant (*p* < 0.01) difference between daytime and nighttime estimates of *k*_2_ (Fig. 2; Table 2). The average daytime *k*_2_ (*k*_day_) was 0.331 h^−1^ and about 2 times higher than *k*_2_ at night (*k*_night_). Similar conditions were observed at GA (Fig. 3; Table 2), suggesting that diel changes in temperature and wind could partly explain the observed difference between day and night variability in *k*_2_ at both sites.

**Table 3:** Estimates of *k*_2_ using the nighttime regression method (*k*_NR_). Values are reported with the respective regression coefficient (*R* ^2^) for each site. The associated plots are available in the Supporting Information (Fig. 3). The temperature‐normalized values of *k*_NR_ for 20°C (*k*_NR(20°C)_), are computed based on the mean nighttime temperature at each site, while the daily NR‐based *k* scaled for a Schmidt number of 600 (*k*_NR(600)_, in m h^−1^) was computed for the mean daily temperatures.

Site	*k*_NR_ [h^−1^] (*R* ^2^)	*k*_NR(20°C)_ [h^−1^]	*k*_NR(600)_ [m h^−1^]	Night temperature (°C)
CE	0.554 (0.78)	0.694	0.273	10.5
GA	0.421 (0.78)	0.539	0.304	8.7

### Dynamics and variability of O_2_ gas exchange

#### *Temperature*

Gas exchange coefficient (*k*_2_) and gas transfer velocity (*k*) are expected to be related to local stream temperature (Elmore and West 1961; Kilpatrick et al. 1989; Demars and Manson 2013). Yet, temperature has been reported to be a weak predictor of *k* in some settings (Tobias et al. 2009; Demars and Manson 2013), as well as in modeling studies (e.g., Correa‐Gonzalez et al. 2014). For example, Tobias et al. (2009) used a modified sulfur hexafluoride (SF_6_) tracer approach to assess variability in *k* (*k*_SF6_) on time scales of hours (in 3 h intervals) and found that *k*_SF6_ varied by 30% over a 32 h observational period and could apportion 39% of the observed variability to changes in temperature (Demars and Manson 2013).

At CE, our hourly estimates of *k* varied by 60%, on average, between consecutive measurements during our 3 d observational period, with temperature accounting for 46% of that variability (Supporting Information Fig. S4). As expected from previous studies (e.g., Tobias et al. 2009; Demars and Manson 2013), part of this variability could be accounted for by applying the Elmore and West (1961) temperature correction to normalize *k* to 20°C (*k*_20_; Supporting Information Fig. S4). However, we found that temperature corrected *k*_20_ could only explain 10% of the *k*–temperature relationship, and only 5% of the variability measured in *k* at CE. This provided further evidence that the dynamics of *k* could not be properly accounted for by simple functions for temperature corrections.

**Figure 4:**
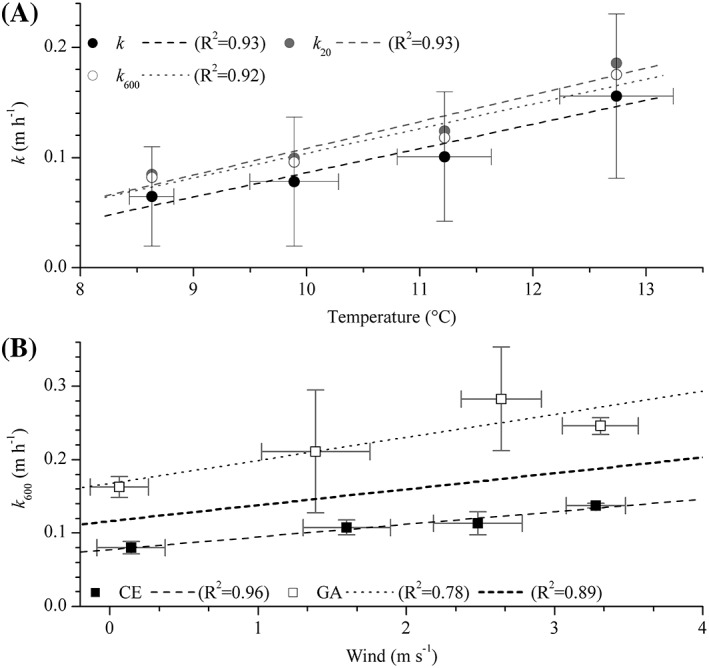
Temperature and wind dependencies of the gas transfer velocity *k*. (**A**) Temperature relationship of *k* at CE. Values for *k* (solid black circles), *k* normalized to 20°C (*k*_20_; open gray circles) and to a Schmidt number of 600 (*k*_600_, solid gray circles) were clustered into 1.5°C temperature increments to account for temporal misalignments and to provide the most robust linear analysis (*R* ^2^ > 0.9; *see* Supporting Information Fig. S4). (**B**) Standardized *k*_600_ as a function of wind speed for CE (solid black squares), GA (open white squares) and both sites combined (dotted line). Wind speeds were clustered into 1 m s^−1^ bins to account for the dominant magnitude of wind fluctuations (*see* Supporting Information Fig. S5). Linear trends were obtained via least squares regression.

Instead, at CE we found that the relationship between *k* and temperature could be strengthened by clustering *k* values to fixed temperature increments, increasing stepwise from 0.5°C to 1.7°C. The best linear relationships (i.e., highest regression coefficients) were observed by clustering data in increments of 1.0–1.5°C (*R* ^2^ > 0.8), with *R* ^2^ decreasing, either side, for shorter or longer increments (Supporting Information Fig. S4) and we found the best fit to the data occurred with 1.5°C increments (Fig. 4A). As the clustering procedure partially accounts for the variability in both temperature and *k*, the trend in increasing fit to the data (higher *R* ^2^) suggested that dynamics in *k* and temperature, and their interaction, may occur at variable time scales. This trend was, however, not observed at GA, where temperature revealed no direct correlation with *k* on hourly time scales (*R* ^2^ = 0.02) or with temperature clustering (*R* ^2^ = 0.03), indicating that temperature changes in the water column did not significantly contribute to *k* variability at the site.

Temperature dynamics, however, may affect gas exchange without any detectable changes in bulk O_2_ concentrations in the water, as recently proposed by Berg and Pace (2017). The authors applied the same AEC approach as in this study, but in an “upside‐down” configuration, with AEC measurements being performed near the (underside) of the stream surface (~ 5 cm below the atmosphere–water interface), rather than near the streambed. This enabled the quantification of O_2_ flux and heat flux across the stream surface, from which they inferred local O_2_ gas exchange and values for *k*. Their results showed that temperature fluctuations occurring just below the surface might bias the quantification of O_2_ fluctuations and thus AEC‐based assessments of *k*. The implications of the study by Berg and Pace (2017) suggest that other methodological approaches commonly used for the quantification of *k* at timescales ranging from minutes to complete diel cycles could also be impacted. The absence of any relationship between water column temperature and *k* at GA could thus, in part, be attributed to small‐scale temperature dynamics that are not accounted for by simple temperature correction functions; although other physical parameters such as wind, for example, could also modulate *k* at GA.

#### *Wind*

Here the reaches were open to the wind (e.g., Supporting Information Fig. S1), with frequent wind‐induced ripples being observed during the day, suggesting an interaction between flow and wind that could affect gas exchange. On time scales of hours, however, wind dynamics revealed no significant relationship with the derived variability in *k* (*R* ^2^ = 0.13). Similar results have also been reported from other methodological approaches, e.g., tracer release (Tobias et al. 2009) and AEC (Berg et al. 2017); although both studies relied on off‐site, rather than on‐site wind measurements. As large variability in both *k* and wind might mask any general trend, wind data were temporally clustered to investigate any time lag between wind dynamics and derived *k* values. Using a bin interval of 1 m s^−1^, which represented the dominant (> 90%) wind fluctuations at each site (Supporting Information Fig. S5), we revealed a robust positive linear relationship (*R* ^2^ > 0.7) between local wind and *k*_600_ at each site and for both sites combined (Fig. 4B). Given the relatively low range in wind speed (0–4 m s^−1^), a linear relationship provided a suitable approximation, although for more extensive ranges in wind speed a quadratic form is more appropriate (e.g., Wanninkhof 1992; Nightingale et al. 2000; Sweeney et al. 2007; Wanninkhof 2014). Similarly to the effect of temperature, increasing or decreasing the size of the wind clustering increment resulted in a lowering of the regression coefficient, thus further supporting the argument of a temporal misalignment between the drivers of *k* variability and actual *k* dynamics.

## *Discussion*

### Parametrization of gas exchange coefficients

Our study was performed under stable discharge and constant stream depth, which allowed both (1) the evaluation of our local O_2_ budget (OB) method for deriving *k*_2_ directly and then, (2) the comparison with the nighttime regression (NR) method and standardized functional equations for *k*_2_ quantification (Table 4). The advantage of the OB method over the latter approaches is that the method is not limited to stable hydrological conditions or specific time periods. This contrasts with the NR method, for instance, where estimates of *k*_2_ obtained at night might not be applicable during the day. Furthermore, the OB approach relies on in situ measurements of local O_2_ mass balance variables, rather than parametrizations and scaling relationship from literature values. The method is therefore ideal for investigating discrepancies between the various procedures for estimating *k*_2_ and thus strengthens the parameterization procedures applied for upscaling global estimates of outgassing coefficients for other gases such as CO_2_ and CH_4_.

**Table 4:** Estimates of *k*_600_ (in m h^−1^) from established empirical equations applied to the CE and GA study sites. Input variables^a^: *d*, stream depth (m); *u*, flow velocity (m s^−1^); *u* ^*^, friction velocity (m s^−1^); *s*, stream slope (m m^−1^); *Q*, discharge (m^3^ s^−1^); and $\mathrm{Fr}=u/\sqrt{\mathrm{gd}}$, Froude number with *g* being the gravitational acceleration constant. Note that most equations provide *k*_2_ at 20°C (*k*_2(20°C)_, in day^−1^; Supporting Information Table S1); *k*_2(20°C)_ values that were scaled to *k*_600_ based on Eq. 7 and the O_2_ Schmidt number for 20°C (Table 1).

Eq.	Reference	Abbr.^b^	Equations	CE	GA
				*k*_600_ [m h^‐1^]	
1	O'Connor and Dobbins (1958)	OD	$k_{2\left(20\;{}^{\circ}C\right)}=3.9\frac{u^{0.5}}{d^{1.5}}$	0.099	0.114
2a	Churchill et al. (1962)	E_4_	$k_{2\left(20\;{}^{\circ}C\right)}=0.0217\frac{u^{2.695}}{d^{3.085}s^{0.825}}$	0.009	0.023
2b		CEB	$k_{2\left(20\;{}^{\circ}C\right)}=5.01\frac{u^{0.969}}{d^{1.673}}$	0.067	0.096
3	Krenkel and Orlob (1963)	E_6_, KO	$k_{2\left(20\;{}^{\circ}C\right)}=173.01\frac{{\left(\mathrm{us}\right)}^{0.404}}{d^{0.66}}$	0.199	0.285
4a	Owens et al. (1964)	E_8_	$k_{2\left(20\;{}^{\circ}C\right)}=6.91\frac{u^{0.73}}{d^{1.75}}$	0.148	0.180
4b		E_9_, OEG	$k_{2\left(20\;{}^{\circ}C\right)}=5.35\frac{u^{0.67}}{d^{1.85}}$	0.139	0.158
5	Dobbins (1965)	DB^e^	$k_{2\left(20\;{}^{\circ}C\right)}=55.2\frac{1+{\mathrm{Fr}}^{2}}{{\left(0.9+\mathrm{Fr}\right)}^{1.5}}\frac{{\left(\mathrm{us}\right)}^{0.375}}{d}\coth\left[\frac{4.75{\left(\mathrm{us}\right)}^{0.125}}{{\left(0.9+\mathrm{Fr}\right)}^{0.5}}\right]$	0.117	0.139
6a	Langbein and Durum (1967)	E_7_	$k_{2\left(20\;{}^{\circ}C\right)}=5.14\frac{u}{d^{1.33}}$	0.048	0.078
6b		LD	$k_{2\left(20\;{}^{\circ}C\right)}=5.14\frac{u}{d^{0.67}}$	0.026	0.054
7	Issacs and Gaudy (1968)	IG	$k_{2\left(20\;{}^{\circ}C\right)}=4.76\frac{u}{d^{1.5}}$	0.051	0.080
8	Cadwallader and McDonnell (1969)	E_3_, CM	$k_{2\left(20\;{}^{\circ}C\right)}=186.07\frac{{\left(\mathrm{us}\right)}^{0.5}}{d}$	0.136	0.184
9	Negulescu and Rojanski (1969)	NR	$k_{2\left(20\;{}^{\circ}C\right)}=10.91{\left(\frac{u}{d}\right)}^{0.85}$	0.085	0.150
10	Thackston and Krenkel (1969)	TK^e^	$k_{2\left(20\;{}^{\circ}C\right)}=24.9\frac{u^{*}\left(1+{\mathrm{Fr}}^{0.5}\right)}{d}$	0.013	0.025
11	Padden and Gloyna (1971)	PG	$k_{2\left(20\;{}^{\circ}C\right)}=4.53\frac{u^{0.703}}{d^{1.054}}$	0.055	0.082
12a	Bennett and Rathbun (1972)	E_1_, BR1	$k_{2\left(20\;{}^{\circ}C\right)}=32.69\frac{u^{0.413}s^{0.273}}{d^{1.408}}$	0.163	0.183
12b		E_2_, BR2	$k_{2\left(20\;{}^{\circ}C\right)}=5.58\frac{u^{0.607}}{d^{1.689}}$	0.140	0.161
13	Parkhurst and Pomeroy (1972)	PP^e^	$k_{2\left(20\;{}^{\circ}C\right)}=48.5\frac{\left(1+0.17{\mathrm{Fr}}^{2}\right){\left(\mathrm{us}\right)}^{0.375}}{d}$	0.097	0.122
14	Bansal (1973)	BN	$k_{2\left(20\;{}^{\circ}C\right)}=1.81\frac{u^{0.6}}{d^{1.4}}$	0.036	0.045
15	Owens (1974)	–	$k_{2\left(20\;{}^{\circ}C\right)}=50.8\frac{u^{0.67}}{d^{0.85}}$ c	0.236	0.373
16	Tsivoglou and Neal (1976)	E_10_, TN	*k*_2(20 ° C)_ = *k*_2_^′^*us* d	0.128	0.325
17	Smoot (1988)	SM	$k_{2\left(20\;{}^{\circ}C\right)}=543\frac{u^{0.5325}s^{0.6236}}{d^{0.7258}}$	0.136	0.206
18	Thackston and Dawson (2001)	TD^e^	$k_{2\left(20\;{}^{\circ}C\right)}=4.97\frac{u^{*}\left(1+9{\mathrm{Fr}}^{0.25}\right)}{d}$	0.012	0.024
19a	Raymond et al. (2012)	Rm_1_	*k*_600_ = 5037(*us*)^0.89^*d*^0.54^	0.113	0.231
19b		Rm_2_	*k*_600_ = 5937(1 − 2.54*Fr*^2^)(*us*)^0.89^*d*^0.58^	0.125	0.252
19c		Rm_3_	*k*_600_ = 1162*u*^0.85^*s*^0.77^	0.096	0.160
19d		Rm_4_	*k*_600_ = 951.5(*us*)^0.76^	0.097	0.154
19e		Rm_5_	*k*_600_ = 2841*us* + 2.02	0.127	0.162
19f		Rm_6_	*k*_600_ = 929(*us*)^0.75^*Q*^0.011^	0.102	0.160
19g		Rm_7_	*k*_600_ = 4725(*us*)^0.86^*Q*^−0.14^*d*^0.66^	0.128	0.287

a Average stream depth and flow velocity are taken from (Table 1). The average shear velocity, u_*_, computed as u_*_ = *u*(*C*_D_)^1/2^, with *C*_D_ being the drag coefficient (Wüest and Lorke 2003). An average *C*_D_ of 3.3 × 10^−3^ was used for both sites based on further surveys of the River Avon sub‐catchments (Rovelli et al. 2017). The average slope, 0.002 m m^−1^, was estimated from GPS measurements during the respective field campaigns.

b Reference equation numbers and abbreviations from Aristegi et al. (2009) and Palumbo and Brown (2014), respectively.

c This equation requires depth in cm and flow velocity in cm s^−1^.

d With *k*_2_′ being 31,183 s m^−1^ d^−1^ for *Q* < 0.280 m^3^ s^−1^ and 22,500 s m^−1^ d^−1^ for *Q* > 0.280 m^3^ s^−1^, respectively (Palumbo and Brown 2014).

e These equations were identified to be the most suited (i.e., top performer) for the site mean depth and flow based on the suggestions of Palumbo and Brown (2014).

**Figure 5:**
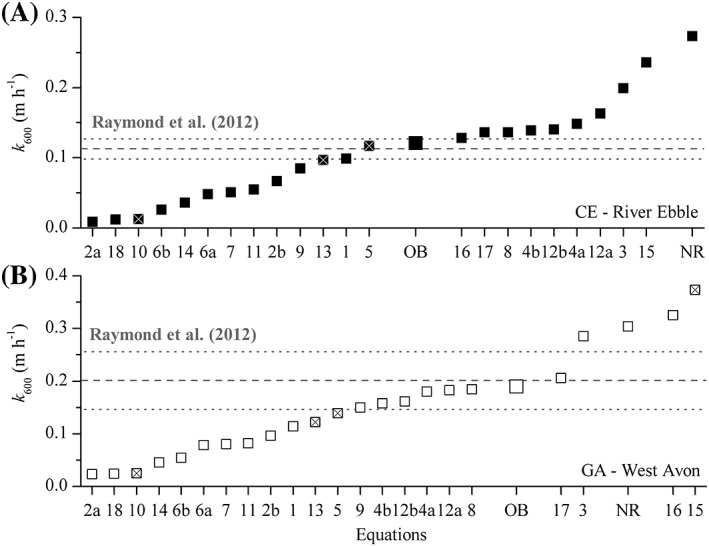
Estimates of *k*_600_ (in m h^−1^) for (**A**) River Ebble and (**B**) West Avon. Estimates are obtained from different published hydrological parameterizations (Eqs. (1)–18, and Raymond et al. (2012)); from the O_2_ budget (OB) method used in this study (*see* Eq. (4)); and from the nighttime regression (NR) method. Equation numbering follows Table 4. Note that crossed equations were reported to be the “top‐performer” within a specific mean depth and mean flow range from an extensive database of tracer‐based *k*_2_ values and hydrological parameters (*see* Palumbo and Brown 2014). Mean value and uncertainty range (dashed and dotted lines) for the hydrological parameterizations of Raymond et al. (2012) were obtained by combining mean and range from of each equation (*see* Table 4; Supporting Information Fig. S6). Note that to better highlight the differences between the OB, NR and common parametrizations, the equation order was re‐arranged to show an incremental increase in *k*_600_.

In this study, the NR method systematically provided among the highest estimates of *k*_2_ for both reaches. Values of *k*_NR_ were, on average, a factor of two higher than *k*_2_ derived by the OB method (Fig. 5). The largest discrepancies, by up to a factor of three, were observed at nighttime, while, during the daytime, mean values were within reported uncertainties for this approach (Palumbo and Brown 2014). Potential issues with NR method applicability or dataset quality were deemed marginal, as both CE and GA met the requirements of the NR method (*see* “Methods” section), and the O_2_ time series were of a high quality (Supporting Information Fig. S3). Therefore, the observed discrepancies in *k*_2_ are attributed to a biased estimation of *R* derived at nighttime using the NR method (*R*_NR_), leading to a systematic overestimation of *k*_2_.

Studies investigating the effect of local inflows of O_2_‐depleted groundwater have shown that groundwater might bias the quantification of *R* in open water approaches and thus whole‐stream O_2_ budget assessments (e.g., McCutchan et al. 2002; Hall and Tank 2005; Koopmans and Berg 2015). The magnitude of such effect, which would lead to an overestimation of *R*, might vary according to hydrological characteristics and hydrological connectivity of each stream, ranging from negligible to substantial (*see* McCutchan et al. 2002). In this study, the contribution from groundwater to the O_2_ budgets was quantified directly. At gaining reach GA, groundwater contributions to whole‐stream estimates of *R* were limited to 0.5 mmol m^−2^ h^−1^, representing < 5% of the mean *R*_NR_ (15.5 mmol m^−2^ h^−1^); the combined *R* from the benthic compartment and water column activity was 6.7 mmol m^−2^ h^−1^. At losing reach CE, contributions from groundwater were negligible but the NR method still estimated *R* at 8.7 mmol m^−2^ h^−1^_,_ which is some threefold higher than our direct measurements (2.8 mmol m^−2^ h^−1^).

An overestimation of *R* during night could also result from a temporal misalignment between actual changes in whole stream respiration and physical parameters controlling gas exchange. As shown in both datasets (Figs. 2, 3), temperature decreased at night until about 6–8 a.m., and directly affected the measured saturation deficit. If *k*_2_ is assumed to be constant, as shown from our NR regression plots (*see* Supporting Information Fig. S3), then the temperature‐driven increase in the O_2_ concentration at saturation, and associated O_2_ saturation deficit, will result in an erroneous estimate of the magnitude of *R*_NR_.

It has recently been shown that *k* (and thus *k*_2_) might display changes of up to a factor of 2–3 over timescales of 10 s of minutes to hours, even under constant hydrological conditions (e.g., Berg and Pace 2017), implying that *k*_2_ will not remain constant over the several hours’ time scale associated with the NR method. Since the NR approach considers changes in the magnitude of *k*_2_ as a direct and instantaneous consequence of changes in *R*, *v*ariability in *k*_2_ could explain the magnitude of the *R*_NR_ bias obtained in this study, although the drivers of that variability, such as temperature, wind and stream discharge, need further investigation (Berg and Pace 2017).

Hydrological parameters such as stream depth, stream flow, and stream energy (e.g., slope, shear velocity) have been used to derive *k*_2_ (e.g., O'Connor and Dobbins 1958; Palumbo and Brown 2014) and subsequently *k*_600_. Given the local hydrology (0.4–0.57 m mean depth, 0.18–0.33 m s^−1^ mean flow velocity), our mean values of *k*_600_ derived with the (OB) method (0.121 ± 0.007 m h^−1^ for CE and 0.190 ± 0.016 m h^−1^ for GA), are well within the range predicted by 19 sets of proposed functional equations extracted from the literature (0.009–0.236 m h^−1^ at CE and 0.023–0.373 m h^−1^ at GA; Table 4; Fig. 5). NR‐derived values were also within that range, although they ranked toward the upper end of the range. As expected from previous studies (*see* Aristegi et al. 2009; Palumbo and Brown 2014), estimates from each equation were highly scattered throughout the obtained range, with little consistency between the two sites. For instance, even equations that were previously identified to perform well in hydrology settings similar to our study, i.e., “top performer” equations (*see* Palumbo and Brown 2014), still showed large discrepancies—both within and across sites (Fig. 5). In contrast, we found that the revised functional equations provided by Raymond et al. (2012) provided well‐constrained estimates, which were not only consistent within sites (*see* Supporting Information Fig. S6), but also between sites, with mean *k*_600_ values (0.113 m h^−1^ for CE and 0.201 m h^−1^ for GA) within 5–7% of the local estimates from our OB method (Fig. 5).

## *Comments and recommendations*

The aquatic eddy co‐variance technique (AEC) has been shown to be an effective tool for quantifying streambed metabolism and, more recently, for the quantification of O_2_ gas exchange and *k* from O_2_ fluxes near the stream surface (Berg and Pace 2017). A combination of the AEC technique with other traditional methods appears to be a promising next step toward better constrained assessments of gas exchange and *k* dynamics in headwaters, provided that careful consideration is given to (1) the site selection and (2) the representativeness of the reach within the km‐sized integration of, e.g., open‐water approaches. The O_2_ budget (OB) approach presented in this study serves as a proof‐of‐concept toward this goal. While the approach is more time consuming and field demanding than traditional methods, it does enable (1) the compartmentalization of stream metabolism and (2) the assessment of short‐term *k* variability. The study has highlighted short‐term variability in *k* dynamics with a dampened relationship to variations of well‐known physical drivers. The apparent lack of correlation could be attributed to temporal misalignments between the variability of the derived *k* and physical drivers, and to small‐scale variability in temperature. This was clearly observed at CE, where temperature‐corrected *k*_20_ and *k*_600_ values still revealed a marked temperature dependency, suggesting that common temperature corrections were insufficient to fully account for the observed *k* variability. It remains unclear whether and to what extend such small‐scale variability in temperature will affect the overall gas exchange.

Although the above aspects are still poorly constrained at the local scale and on short time scales (hours), estimates of *k* from the most recent functional parametrizations compared well with the independent assessments of this study and should therefore be preferred over earlier parametrizations. Validations of *k* on local scales such as the ones presented in this study are strongly required to strengthen and add more confidence to the upscaling of *k* for the quantification of large‐scale metabolism and global emission of climate‐relevant gases such as CO_2_ and CH_4_ across headwaters and throughout riverine networks.

## Conflict of Interest

None declared.

## Supporting information

Appendix S1: Supporting information

## Endnotes

1 Reach designations within the Macronutrient Cycles Programme (https://nerc.ukri.org/research/funded/programmes/macronutrient/).

2 Schmidt number for CO_2_ at 20°C and O_2_ at 17.5°C in freshwater.
