# Supplementary material for: Headwater gas exchange quantified from O2 mass balances at the reach scale
Source: Limnol Oceanogr Methods. 2018 Sep 28;16(10):696–709. doi: 10.1002/lom3.10281 (PMC6220971; doi:10.1002/lom3.10281)
Supplement: Supplementary file 1 — Appendix S1: Supporting information [file LOM3-16-696-s001.docx]

**Supporting information for**

**Headwater gas exchange quantified from O_2_ mass balances at the reach scale**

L. Rovelli, ^1,2,*^ K. M. Attard, ^2,3^ C. M. Heppell, ^4^ A. Binley, ^5^ M. Trimmer, ^6^ and R. N. Glud, ^1,^^7^

^1^ Scottish Marine Institute, Scottish Association for Marine Sciences, PA37 1QA Oban, United Kingdom

^2^ Nordcee, Department of Biology, University of Southern Denmark, 5230 Odense M, Denmark

^3^ Tvärminne Zoological Station, University of Helsinki, 10900 Hanko, Finland

^4^ School of Geography, Queen Mary University of London, Mile End Road, E1 4NS London, United Kingdom

^5^ Lancaster Environment Centre, Lancaster University, LA1 4YQ Lancaster, United Kingdom

^6^ The School of Biological and Chemical Sciences Queen Mary University of London, E1 4NS London, United Kingdom

^7^ Department of Ocean and Environmental Sciences, Tokyo University of Marine Science and Technology, 4-5-7 Konan, Minato-ku, 108-8477 Tokyo, Japan

* corresponding author

10 pages, 1 table, 6 figures

**Supporting Tables**

| Table 4. Estimates of *k*_2_ at 20°C (*k*_2(20°C)_, in day^-1^) from established empirical equations applied to the CE and GA study sites. Input variables^#^: *d* – stream depth (m), *u* – flow velocity (m s^-1^), *u*^*^ – friction velocity (m s^-1^), *s* – stream slope (m m^-1^), Q – discharge (m^3^ s^-1^), and $Fr=u/\sqrt{gd}$ – Froude number with *g* being the gravitational acceleration constant. Note that for equations providing *k*_600_ (in m day^-1^) *k*_2(20°C)_ values were scaled based on Eq. 7 and the O_2_ Schmidt number at 20°C. | | | | | |
| --- | --- | --- | --- | --- | --- |
| Eq. | Reference | Abbr.^##^ | Equations | CE | GA |
|  |  |  |  | *k*_2(20°C)_ [day^-1^] | |
| 1 | O'Connor and Dobbins (1958) | OD | $k_{2}=3.9\frac{u^{0.5}}{d^{1.5}}$ | 6.303 | 5.206 |
| 2a | Churchill et al. (1962) | E_4_ | $k_{2}=0.0217\frac{u^{2.695}}{d^{3.085}s^{0.825}}$ | 0.563 | 1.044 |
| 2b |  | CEB | $k_{2}=5.01\frac{u^{0.969}}{d^{1.673}}$ | 4.227 | 4.382 |
| 3 | Krenkel and Orlob (1963) | E_6_, KO | $k_{2}=173.01\frac{{(us)}^{0.404}}{d^{0.66}}$ | 12.658 | 13.010 |
| 4a | Owens et al. (1964) | E_8_ | $k_{2}=6.91\frac{u^{0.73}}{d^{1.75}}$ | 9.407 | 8.226 |
| 4b |  | E_9_, OEG | $k_{2}=5.35\frac{u^{0.67}}{d^{1.85}}$ | 8.825 | 7.201 |
| 5 | Dobbins (1965) | DB* | $k_{2}=55.2\frac{1+{Fr}^{2}}{{(0.9+Fr)}^{1.5}}\frac{{(us)}^{0.375}}{d}\coth\left[ \frac{4.75{(us)}^{0.125}}{{(0.9+Fr)}^{0.5}} \right]$ | 7.443 | 6.342 |
| 6a | Langbein and Durum (1967) | E_7_ | $k_{2}=5.14\frac{u}{d^{1.33}}$ | 3.029 | 3.582 |
| 6b |  | LD | $k_{2}=5.14\frac{u}{d^{0.67}}$ | 1.681 | 2.472 |
| 7 | Issacs and Gaudy (1968) | IG | $k_{2}=4.76\frac{u}{d^{1.5}}$ | 3.264 | 3.650 |
| 8 | Cadwallader and McDonnell (1969) | E_3_, CM | $k_{2}=186.07\frac{\left( us \right)^{0.5}}{d}$ | 8.611 | 8.386 |
| 9 | Negulescu and Rojanski (1969) | NR | $k_{2}=10.91\left( \frac{u}{d} \right)^{0.85}$ | 5.419 | 6.856 |
| 10 | Thackston and Krenkel (1969) | TK* | $k_{2}=24.9\frac{u^{*}\left( 1+{Fr}^{0.5} \right)}{d}$ | 0.813 | 1.140 |
| 11 | Padden and Gloyna (1971) | PG | $k_{2}=4.53\frac{u^{0.703}}{d^{1.054}}$ | 3.473 | 3.758 |
| 12a | Bennett and Rathbun (1972) | E_1_, BR1 | $k_{2}=32.69\frac{u^{0.413}s^{0.273}}{d^{1.408}}$ | 10.357 | 8.365 |
| 12b |  | E_2_, BR2 | $k_{2}=5.58\frac{u^{0.607}}{d^{1.689}}$ | 8.884 | 7.357 |
| 13 | Parkhurst and Pomeroy (1972) | PP* | $k_{2}=48.5\frac{\left( 1+0.17{Fr}^{2} \right){(us)}^{0.375}}{d}$ | 6.140 | 5.590 |
| 14 | Bansal (1973) | BN | $k_{2}=1.81\frac{u^{0.6}}{d^{1.4}}$ | 2.254 | 2.044 |
| 15 | Owens (1974) | – | $k_{2}=50.8\frac{u^{0.67}}{d^{0.85}}$^###^ | 14.998 | 17.013 |
| 16 | Tsivoglou and Neal (1976) | E_10_, TN | $k_{2}=k_{2}'us$^####^ | 8.100 | 14.850 |
| 17 | Smoot (1988) | SM | $k_{2}=543\frac{u^{0.5325}s^{0.6236}}{d^{0.7258}}$ | 8.634 | 9.387 |
| 18 | Thackston and Dawson (2001) | TD* | $k_{2}=4.97\frac{u^{*}\left( 1+{9Fr}^{0.25} \right)}{d}$ | 0.740 | 1.077 |
| 19a | Raymond et al. (2012) | Rm_1_ | $k_{600}={5037(us)}^{0.89}d^{0.54}$ | 7.150 | 10.524 |
| 19b |  | Rm_2_ | $k_{600}={5937(1-2.54{Fr}^{2})(us)}^{0.89}d^{0.58}$ | 7.965 | 11.520 |
| 19c |  | Rm_3_ | $k_{600}=1162{u^{0.85}s}^{0.77}$ | 6.106 | 7.321 |
| 19d |  | Rm_4_ | $k_{600}={951.5(us)}^{0.76}$ | 6.169 | 7.018 |
| 19e |  | Rm_5_ | $k_{600}=2841us+2.02$ | 8.048 | 7.410 |
| 19f |  | Rm_6_ | $k_{600}={929(us)}^{0.75}Q^{0.011}$ | 6.488 | 7.296 |
| 19g |  | Rm_7_ | $k_{600}={4725(us)}^{0.86}Q^{-0.14}d^{0.66}$ | 8.115 | 13.108 |
| #: Average stream depth and flow velocity are taken from (Table 1). The average shear velocity, u_*_, computed as $u_{*}=u{(C_{D})}^{1/2}$, with *C*_D_ being the drag coefficient (Wüest and Lorke 2003). An average *C*_D_ of 3.3 x 10^-3^ was used for both sites based on further surveys of the River Avon sub-catchments (Rovelli et al. 2017). The average slope, 0.002 m m^-1^, was estimated from GPS measurements during the respective field campaigns.  ##: Reference equation numbers and abbreviations from Aristegi et al. (2009) and Palumbo and Brown (2014), respectively.  ###: This equation requires depth in cm and flow velocity in cm s^-1^.  ####: with *k*_2_’ being 31183 s m^-1^ d^-1^ for Q < 0.280 m^3^ s^-1^ and 22500 s m^-1^ d^-1^ for Q > 0.280 m^3^ s^-1^, respectively (Palumbo and Brown 2014).  *: These equations were identified to be the most suited (i.e., top performer) for the site mean depth and flow based on the suggestions of Palumbo and Brown (2014). | | | | | |

**Supporting Figures**


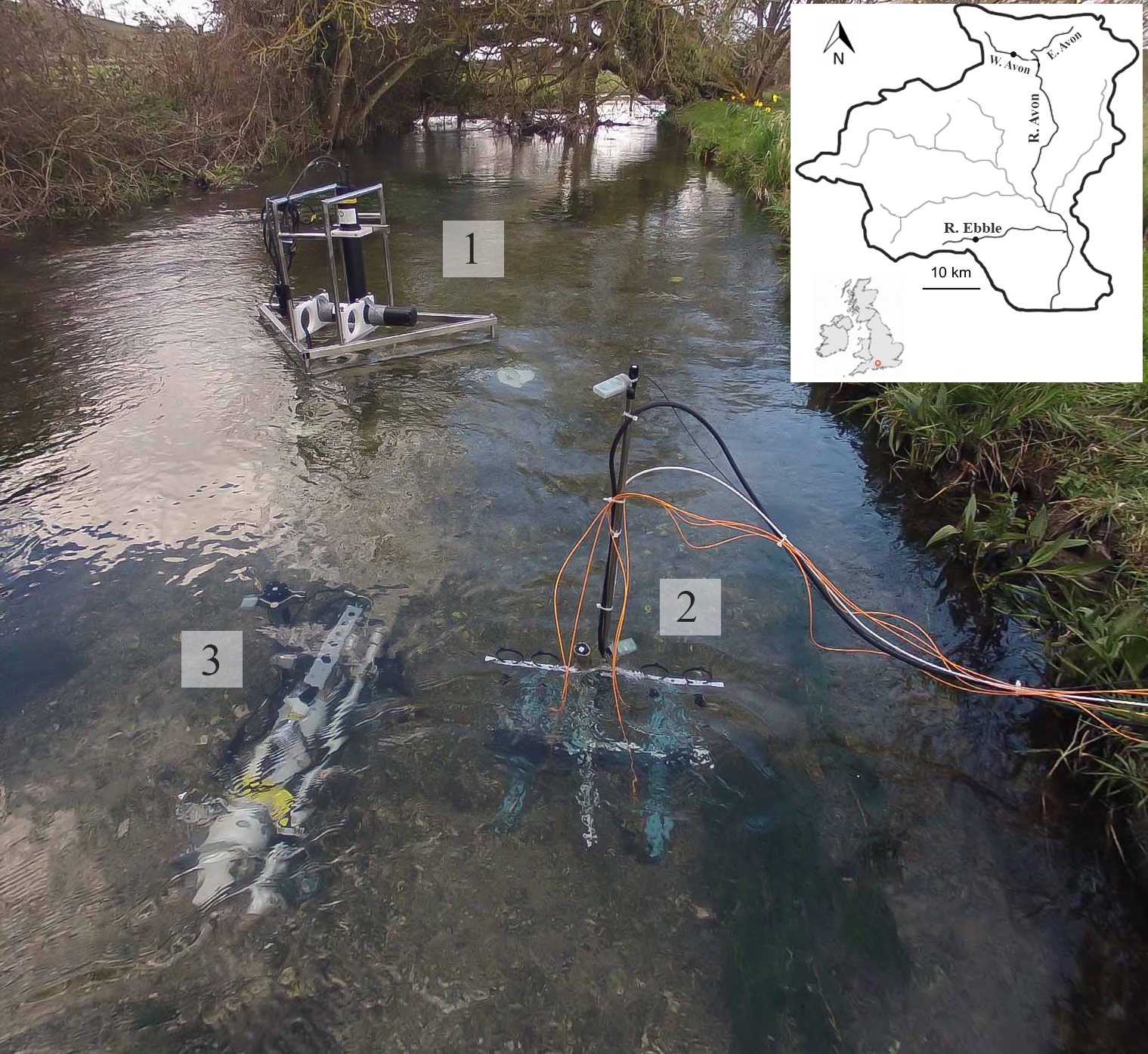


**Figure S1.** Catchment of the Upper River Avon and field measurement setup in the River Ebble (UK). The instruments consisted of [1] an aquatic eddy co-variance module, [2] a water column incubation stand, and [3] a CTD logger, which was also equipped with a PAR sensor and an O_2_ optode. The setup also included riparian piezometers and a meteorological station (*see* Heppell et al., 2017). Dots on the catchment maps indicate the investigated reaches on the rivers Ebble (CE) on the Chalk, and West Avon (GA) on the Greensand.

| 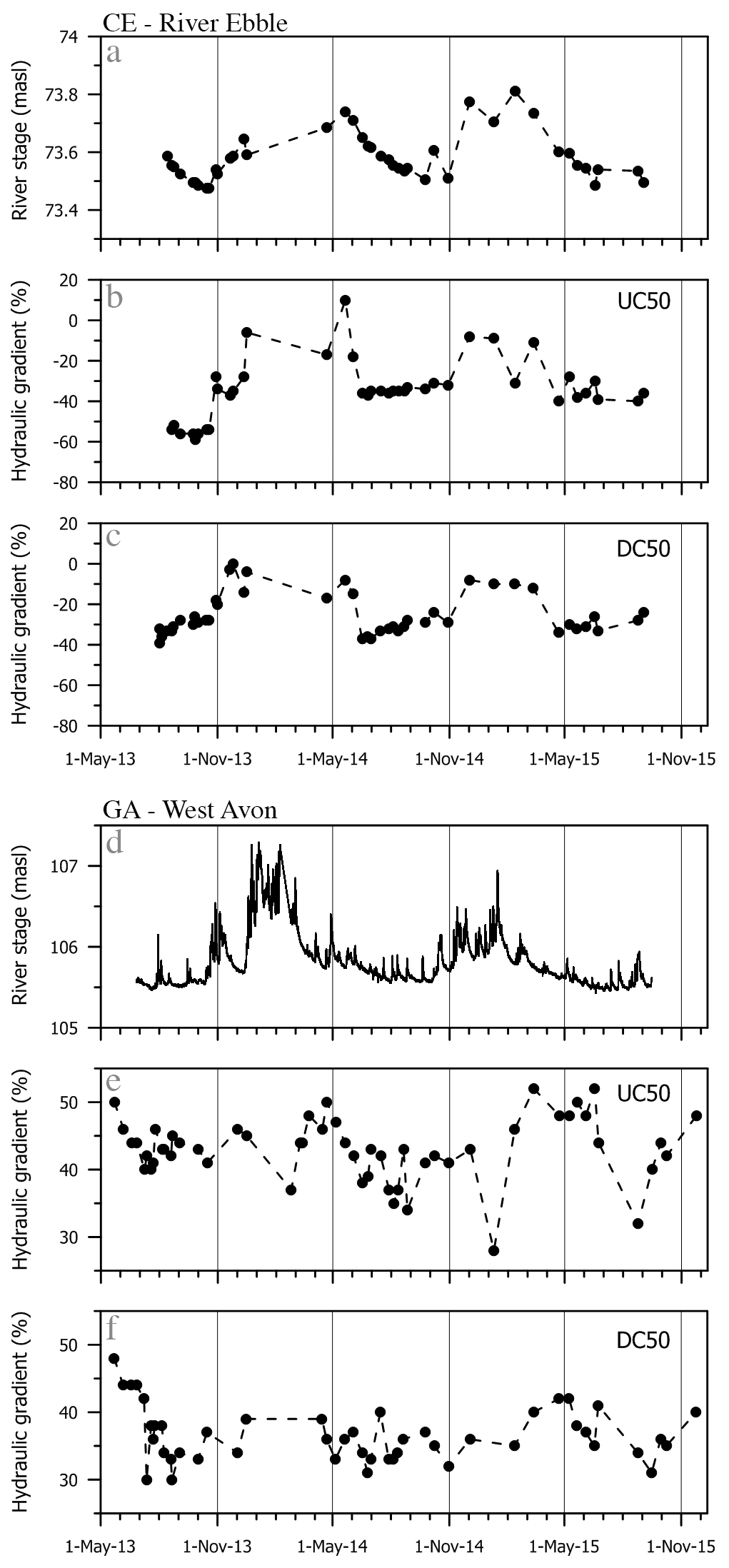 |
| --- |

**Figure S2.** Stream stage and groundwater hydraulic gradient at the rivers Ebble (CE; a–c) and West Avon (GA; d–f). The hydraulic gradient was determined from in-stream 50 cm deep piezometers 10 m apart, upstream (UC50) and downstream (DC50), from dipped levels. Positive gradients indicate groundwater upwelling, i.e. a net inflow of groundwater into the stream, while negative gradients indicate downwelling, i.e., a net loss of water from the stream to the aquifer. Estimates for spring 2013 were assumed to be equal to the same period in 2014, as mean seasonal gradients were found to be comparable across consecutive years. Stage plot for CE (a) are based on dips, while GA stage time series (d) was obtained at 15-min interval from a pressure logger (HOBO pressure transducer; Onset, USA) installed in the piezometer.

**
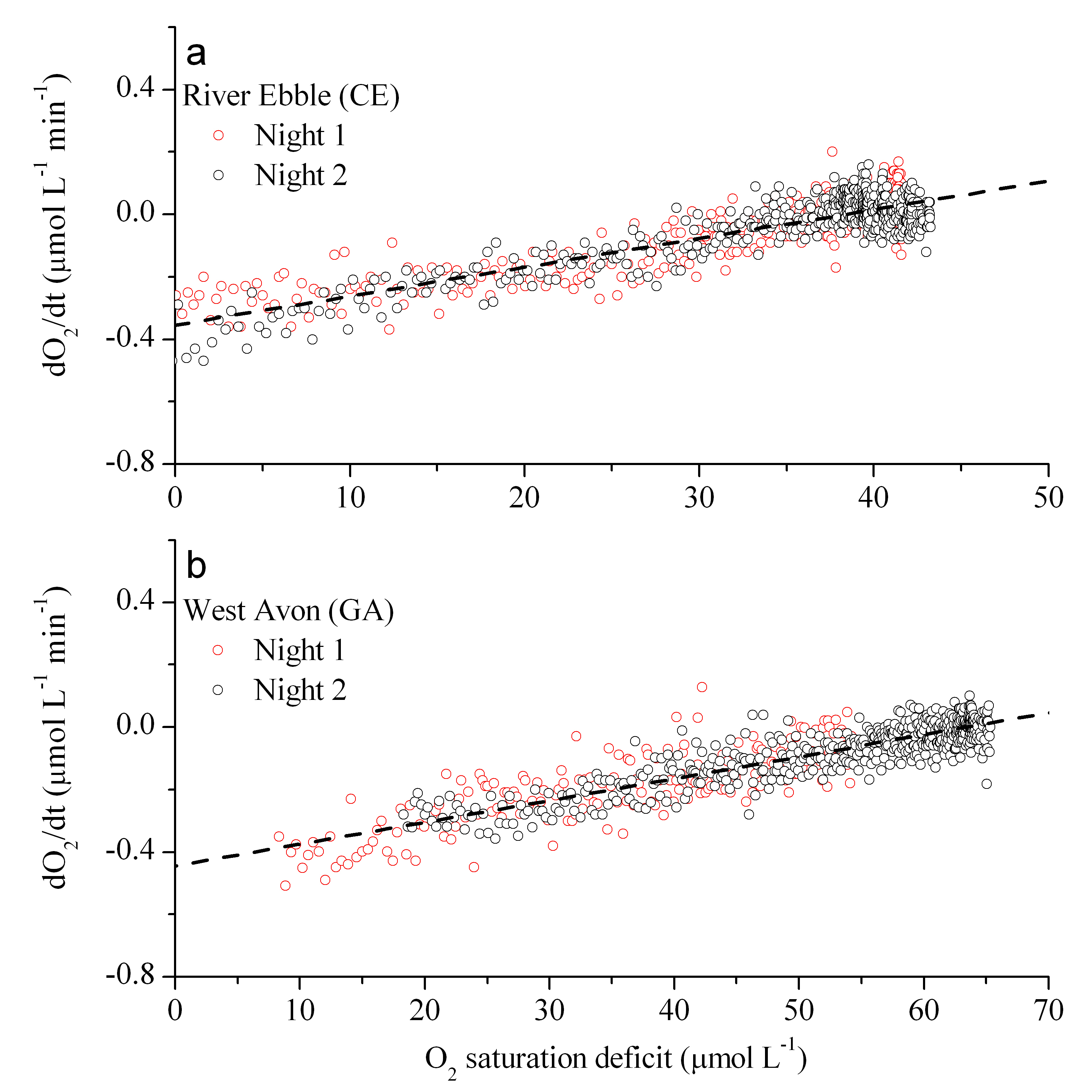
**

**Figure S3.** Nighttime regression (NR) method applied to in-stream measurements of O_2_ at the rivers (a) Ebble (CE) and (b) West Avon (GA). The dashed line indicates the linear regression fitted through both night’s data combined, with the slope representing the nighttime gas exchange coefficient (*k*_NR_) and the intercept representing nighttime respiration. Values of *k*_NR_ and associated regression coefficients, R^2^, are presented in Table 2. Note that no statistically significant (p<0.05) difference was observed between *k*_NR_ from night 1 and 2 at GA, while *k*_NR_ values at CE were statistically different, but within <10%.

**
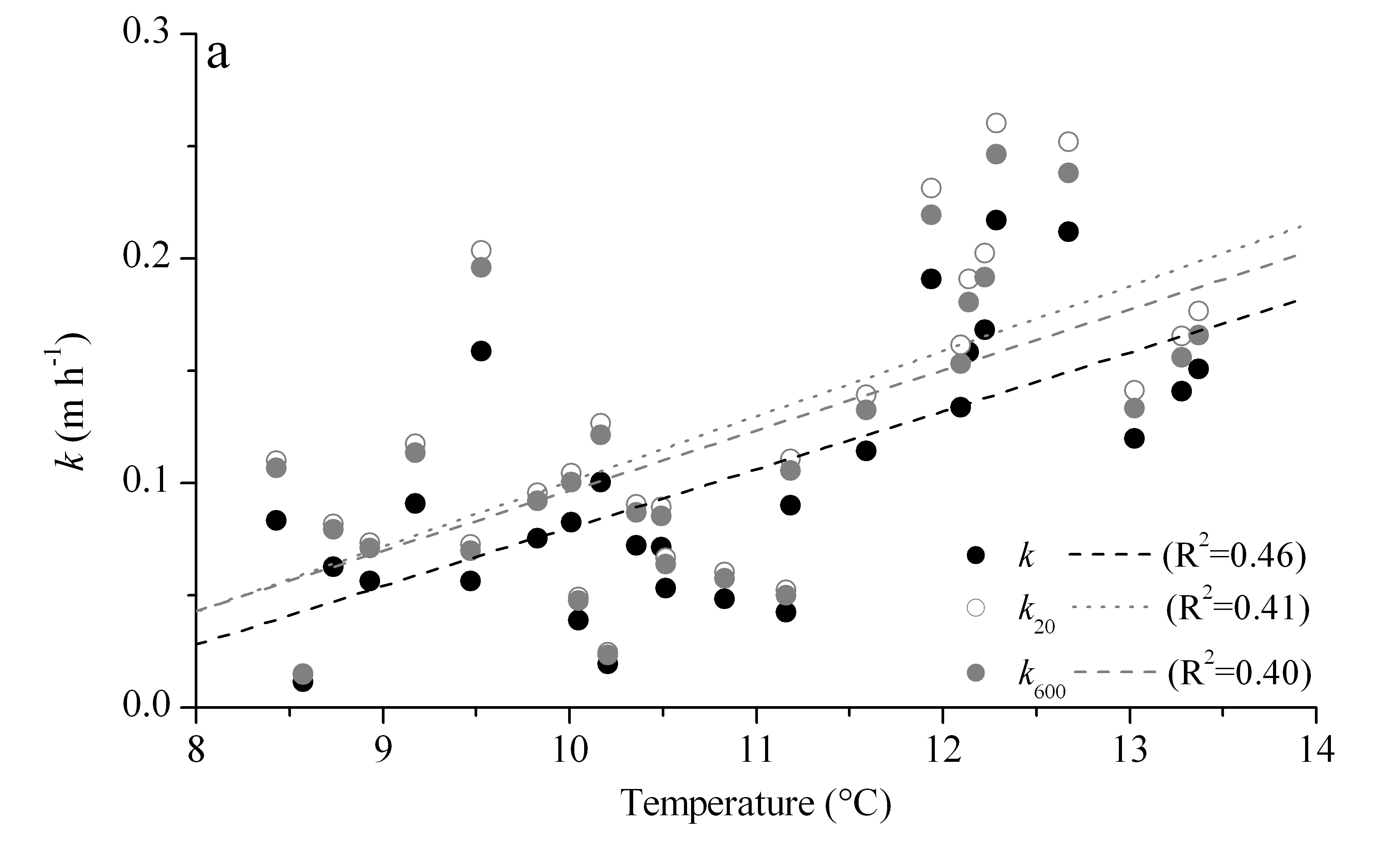

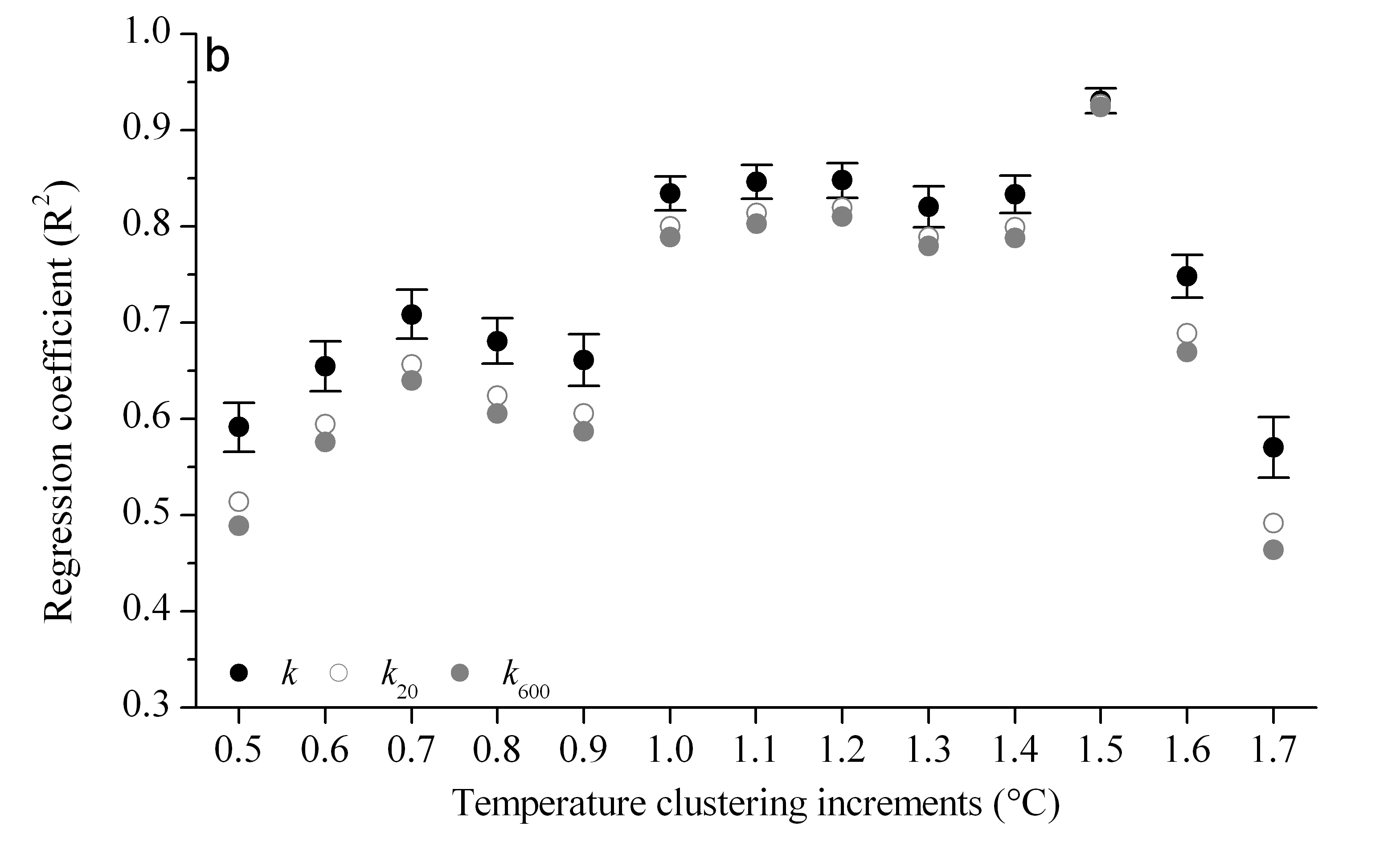
**

**Figure S4.** Temperature dependencies of the gas transfer velocity *k* at river Ebble (CE). (a) Local gas transfer velocity, *k*, and *k* standardized to 20°C (*k*_20_) and to a Schmidt number of 600 (*k*_600_) as a function of temperature. (b) Effect of temperature clustering increments on the relationship between temperature and local oxygen gas transfer velocity, *k*, and for *k* standardized to 20°C (*k*_20_) and to a Schmidt number of 600 (*k*_600_). For each increment, only clusters with *n*≥3 were considered usable for analysis by linear regression, to ensure that sparse single data points or data pairs would not bias the relationship; similarly, only increments with at least 4 data clusters were further considered for the regression analysis. The highest correlation (R^2^>0.9) was obtained by clustering temperature in 1.5 °C increments. The standard deviation bars of R^2^ values were comparable in size for *k*, *k*_20_ and *k*_600_, but are only shown for *k*.

**
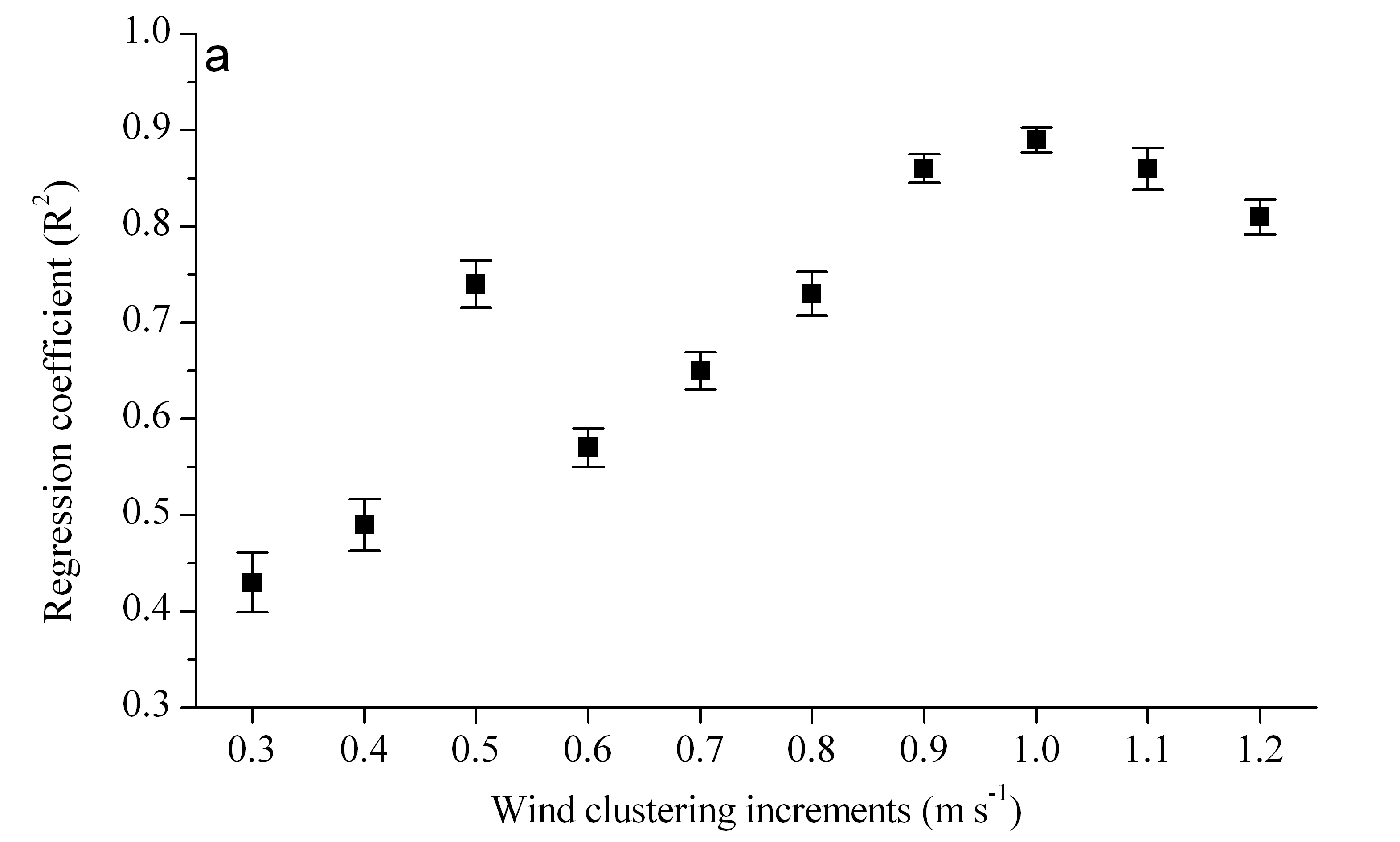

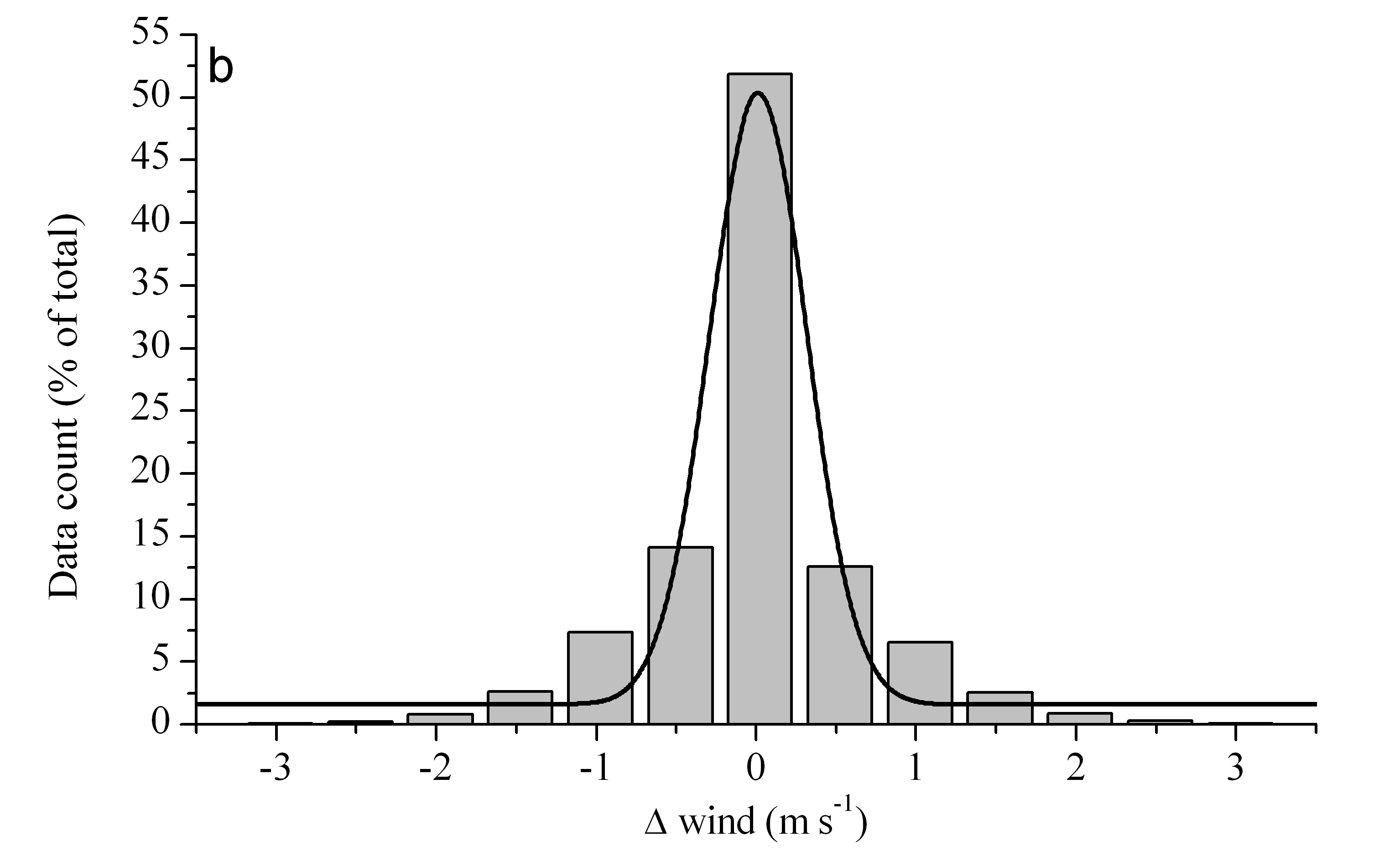
**

**Figure S5.** (a) Effect of wind clustering increments on the relationship between wind speed (m s^-1^) and the standardized oxygen gas transfer velocity to a Schmidt number of 600 (*k*_600_). For each increment, only clusters with *n*≥3 were considered suitable for analysis by linear regression, to ensure that sparse single data points or data pairs would not bias the relationship; similarly, only increments with at least 4 data clusters were further considered for the regression analysis. Note that the highest correlation was obtained by clustering wind dynamics in 1 m s^-1^ increments. Error bars indicate the standard deviation of R^2^. (b) Frequency count analysis on combined wind measurements at river Ebble (CE) and West Avon (GA). Based on a Gaussian distribution curve fit (R^2^=0.97), >90% of the wind fluctuations were within ±1 m s^-1^.

**
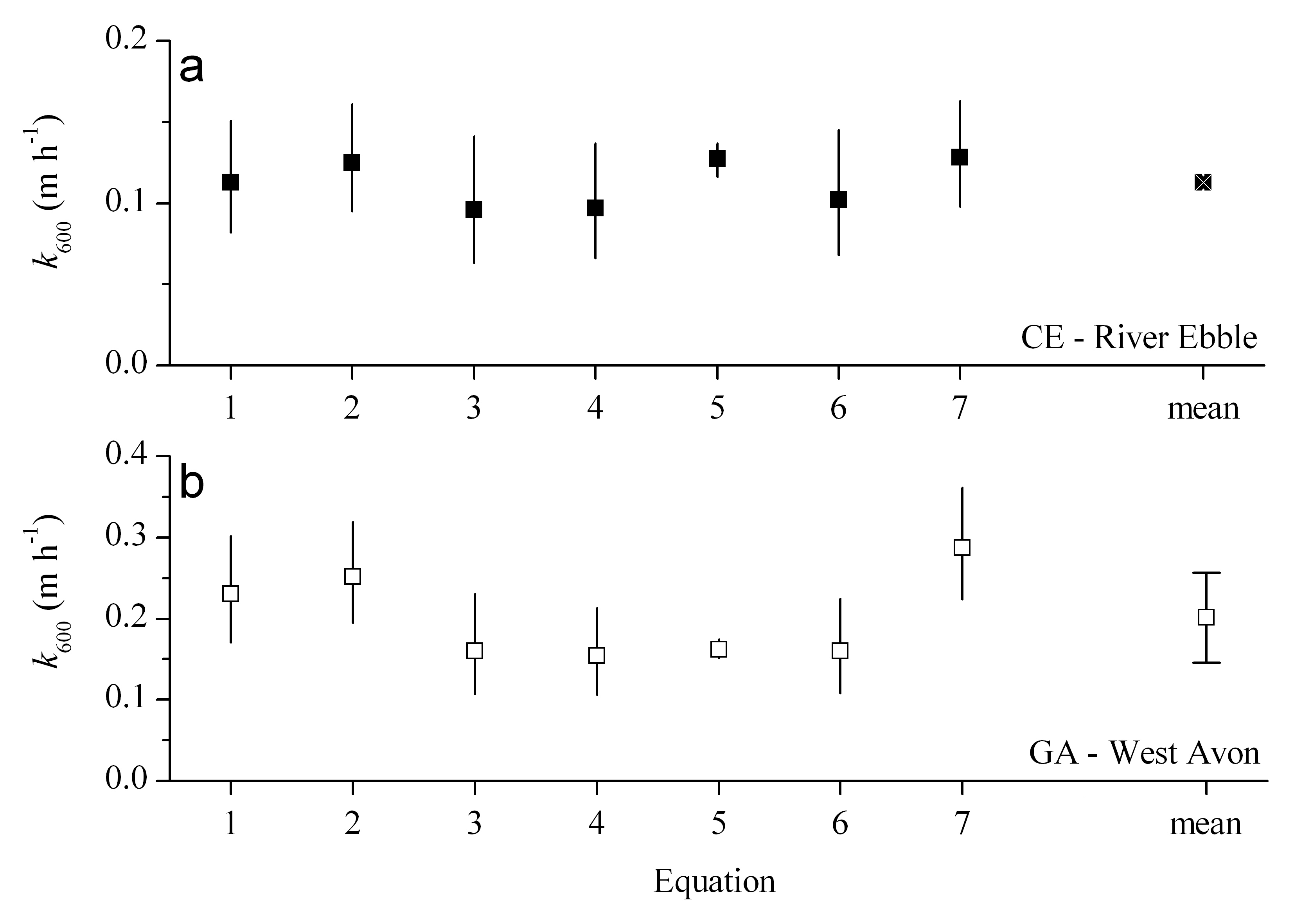
**

**Figure S6.** Prediction of gas transfer velocity (*k*_600_) for (a) River Ebble (CE) and (b) West Avon (GA) based on each streams’ hydraulic properties and the equations provided by Raymond et al. (2012). Error bars on each equation represent the statistical range of the estimates and were obtained by accounting for the variability (i.e., standard deviation) of each parameter (*see* Raymond et al. 2012). The combined mean value ± standard deviation from all equation is also provided for comparison.
